# Perspectives Regarding the Risk of Introduction of the Japanese Encephalitis Virus (JEV) in the United States

**DOI:** 10.3389/fvets.2020.00048

**Published:** 2020-02-07

**Authors:** Ana R. S. Oliveira, Lee W. Cohnstaedt, Leela E. Noronha, Dana Mitzel, D. Scott McVey, Natalia Cernicchiaro

**Affiliations:** ^1^Center for Outcomes Research and Epidemiology, Department of Diagnostic Medicine and Pathobiology, College of Veterinary Medicine, Kansas State University, Manhattan, KS, United States; ^2^Arthropod-Borne Animal Diseases Research, Agricultural Research Service, United States Department of Agriculture, Manhattan, KS, United States

**Keywords:** arbovirus, Japanese encephalitis, JEV, perspective, risk assessment

## Abstract

Japanese encephalitis (JE) is a zoonotic, emerging disease transmitted by mosquito vectors infected with the Japanese encephalitis virus (JEV). Its potential for emergence into susceptible regions is high, including in the United States (US), and is a reason of economic concern among the agricultural community, and to public health due to high morbidity and mortality rates in humans. While exploring the complexities of interactions involved with viral transmission, we proposed a new outlook on the role of vectors, hosts and the environment under changing conditions. For instance, the role of feral pigs may have been underappreciated in our previous work, given research keeps pointing to the importance of susceptible populations of wild swine in naïve regions as key elements for the introduction of emergent vector-borne diseases. High risk of JEV introduction has been associated with the transportation of infected mosquitoes via aircraft. Nonetheless, no JEV outbreaks have been reported in the US to date and results from a qualitative risk assessment considered the risk of establishment to be negligible under the current conditions (environmental, vector, pathogen, and host). In this work, we discuss virus-vector-host interactions and ecological factors important for virus transmission and spread, review research on the risk of JEV introduction to the US considering the implications of risk dismissal as it relates to past experiences with similar arboviruses, and reflect on future directions, challenges, and implications of a JEV incursion.

## Introduction

The Japanese encephalitis virus (JEV) is a flavivirus transmitted by mosquitoes and the most important cause of viral encephalitis in Southeast Asia and the Western Pacific Rim. Affecting around 68,000 people yearly, Japanese encephalitis (JE) is a debilitating disease with no cure, although there is a vaccine available, which is used extensively in most endemic countries. The case fatality risk may approach 25% and up to 50% of the patients that survive can develop debilitating permanent neurological damage (1, 2). Chronic sequelae, including cognitive dysfunction and neurologic deficits, affect mainly children and are responsible for the high burden of disease of JE globally (3, 4).

Viral transmission is influenced by complex interactions that occur among virus, vector and host, and is driven by environmental, genetic, and ecological determinants (5). The enzootic cycle of JEV is maintained by pigs (the main JEV amplifying host) and ardeid birds, with more than 30 mosquito species identified as potential vectors (3, 6–9). Humans are dead-end hosts that do not amplify the virus nor sustain mosquito infection due to low peaks of viremia (3).

Having expanded from Japan, where it was first isolated, JEV has spread to all neighboring countries, now covering most regions in Southeast Asia. Besides the wide distribution of JEV, recent evidence of geographical genotype displacement has pointed to the changing dynamics of JEV transmission, raising public health concern regarding virus spread to susceptible regions of the globe (4, 10–12). Japanese encephalitis virus genetic material has already been identified in mosquitoes and birds collected in northern Italy, where human cases are unreported to date (13, 14); concurrently, other arboviruses have been emerging in previously unaffected areas, with one of the most recent examples being the occurrence of outbreaks of Zika (although humans are reservoir of this virus) virus in South America (15). In the United States (US), specifically, the introduction of the West Nile virus (WNV) has demonstrated the vulnerability for the emergence of exotic pathogens (16). Moreover, the presence of competent vectors and hosts, the apt weather and climatic conditions in most US states, the non-existence of active JEV surveillance programs and cross-reactivity of JEV with other flaviviruses in diagnostic testing, as well as the increased international travel and trade, make the US a suitable region for JEV introduction and spread (7, 16–18).

Geographical expansion of the virus depends on biotic and abiotic factors which are not static; changes in those factors, such as vector and host population abundance, distribution, and composition, can influence forecasted local transmission cycles. Thus, the aim of this article is to: (1) discuss current advances in virus-vector-host interactions and ecological factors important for virus transmission and spread with a review of research addressing the risk of introduction of JEV in the US, and (2) consider future directions, challenges and implications for JEV introduction, including potential surveillance, and vector mitigation strategies.

## Current Advances

### Virus-Vector-Host-Environment Interactions

#### Lessons Learned Regarding Virus-Vector-Host Interactions

Our previous studies focused on the relative role that various vectors and hosts have on the epidemiology of JEV (7–9). Mosquito vectors other than *Culex tritaeniorhynchus* were found to have higher pooled proportions of JEV infection (7, 8), as well as infection and transmission risks (9). To date, *Culex tritaeniorhynchus* has been considered the most important JEV vector in Southeastern Asia (6); however, this may be the result of an overrepresentation of this species in the literature due to issues related to study and sampling design (19). In fact, the highest pooled infection rate estimates were observed in *Culex annulirostris, Culex sitiens*, and *Culex fuscocephala* (9). *Aedes japonicus* has also been identified as a vector with high JEV infection^1^ (90%) and transmission^2^ rates (75%), pointing to its importance as a potential vector species for the spread of JEV to susceptible regions where it is also present, such as the US (21) and Europe (22). Furthermore, reported pooled estimates of JEV transmission risk in *C. tritaeniorhynchus* are as low as 36% (9), which is much lower than estimates for other mosquito species that are not commonly associated with JEV infection or transmission.

Despite being the primary mammalian amplifying host for JEV (6), meta-regression modeling did not identify domestic pigs as the host species with the highest proportion of JEV infection (7). Nonetheless, North American domestic pigs were shown to be susceptible to JEV experimental infection (23–25) and although the majority of pigs in the US are housed indoors, commercial housing does not preclude mosquito exposure (26–28).

Other hosts, including wild pigs [i.e., pigs that have escaped or been released in the wild (GISD)], have greater pooled proportion of infection estimates when compared to domestic pigs (53 vs. 41%) (7)^3^. This could be related to the intensification of industrial pig farming and biosecurity measures, as well as the decrease in backyard pig rearing in Asia (4). Conversely, increasingly higher populations of wild swine have been identified in certain regions of Asia, potentiating the role of these animals in the ecology of JEV (29–32). Wild pigs are known to play a role in the transmission of several disease agents, including JEV (32), and represent a rapidly growing, free-range population of vertebrate hosts that is expanding worldwide (32–36). In the US, this species has expanded to 35 states due to their adaptability to geographic and climatic conditions and the lack of natural predators (37). The potential of wild pigs as reservoirs and drivers of disease is further increased due to their destructive behavior, which has created new mosquito larval habitats (38), and the possible vector-free JEV transmission between pigs (39, 40).

The estimated proportion of JEV infection in ardeid birds such as herons, although lower than in swine, was reported to be 28% (7). In the US, national surveys from 1966 to 2015 showed that some ardeid bird populations are increasing annually (41). This includes ring-bill gulls (*Larus delawarensis)* and great egrets (*Ardea alba*), which are susceptible to JEV under experimental conditions, with virus shedding via oral and cloacal secretions (17). The epidemiological significance of the latter is not yet known, but like the recent evidence of vector-independent transmission in pigs, it highlights fundamental knowledge gaps surrounding JEV transmission.

#### JEV Genomics and Phylogeny

**Figure 1:**
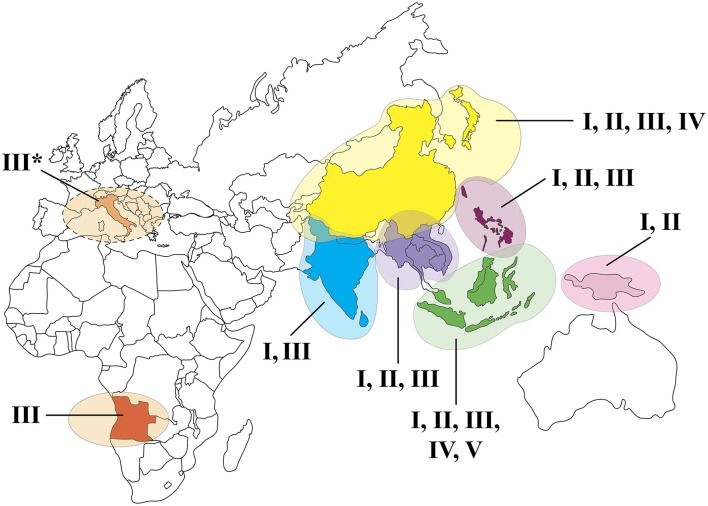
Map of the geographic distribution of JEV genotypes. *No human cases of JE have been reported in Italy to date.

The JEV strains that have been isolated since its discovery can be classified into one of five JEV genotypes [genotype I (GI) to V (GV)] (42). Historically, JEV circulated throughout most of Asia, but various genotypes have spread geographically or have re-emerged in recent years (Figure 1). In 1995, JEV (genotype GII) demonstrated to spread outside of Asia with widespread activity in the Torres Strait of the Australasia region for the first time (43). Approximately 5 years later, a new JEV genotype (GI) was isolated from sentinel pigs and mosquitoes found in the same area as the previous outbreak (44).

Genotype V virus was first isolated in Malaysia in the 1940s and then went undetected until 2009, when it was isolated from a pool of mosquitoes in Tibet and then again in 2010 in the Republic of Korea (45, 46). Genotype V is not a common genotype, with only three isolates having been detected. However, the question arises if the re-emergence after so many years is indicative of genotypic shift in the area.

In addition to the geographic spread, changes in the molecular epidemiology of JEV have occurred throughout Asia. Until the 1990s, GIII was the dominant genotype in Asia, however, surveillance data revealed that GI gradually replaced GIII as the most frequent genotype in many Asian countries. Sequence analysis identified a few variations in the genome that may have played a role in the phenotypic change (42). However, further research is needed to determine if these genetic changes provided an advantage for the virus to survive and thrive in the temperate area. Other studies compared replication efficiency of GI isolates to GIII isolates. Depending on the study, GI was shown to replicate more efficiently in pig, avian, and mosquito cells than GIII (10, 47, 48). Genotype I had a higher infection rate and shorter extrinsic incubation period than GIII during *in vivo* studies using *C. quinquefasciatus* (12). Whereas, these studies help to explain how GI might have displaced the previous genotype, other host and environmental factors, such as effects of immunity of a population to the different genotypes, changes in farming, and animal husbandry practices, and changes in migratory patterns of birds, may have also contributed to the emergence of GI. The recent spread and displacement of JEV demonstrates the importance of understanding how small changes in viral genetics or the introduction of a different strain can lead to an expansion in host range, enhanced vector competence, and hence, arboviral emergence, and increase transmission potential (49).

#### Ecological Factors Important for Viral Transmission and Spread

Emergence of arboviruses frequently follows change in one or various ecological or environmental factors. For JEV, these include precipitation, humidity, temperature, altitude, as well as aspects related to vegetation, land usage, and agricultural practices (5, 50, 51).

Gould et al. (50) discussed the impact of urbanization due to the increase in population densities, which have led to a higher exposure of humans to mosquito vectors and to changes in the interaction patterns occurring among virus, vectors, and hosts. The intensification of deforestation, agriculture, and animal production is the natural response to the pressures of a growing urbanized population. Likewise, the domestication of arthropods in order to adapt to the modern human environment is rampant, as is the invasion of humans into areas that were previously only inhabited by wild flora and fauna, hence changing completely pre-existing dynamics (50). Increased urbanization can also lead to concentration of susceptible human hosts, which depending on their socioeconomic status, can also be conducive to enhanced transmission (49).

Geographic expansion of the virus can result from viral adaptation and displacement. Vector and host population growth and expansion, and improved viral amplification in vertebrate hosts may be related to elongation of seasons, shortening of gonotrophic cycles, and creation of new niches that are associated with environmental changes (e.g., global warming). Invasion and expansion of hosts and vectors through dispersal or migration, are also facilitated by tropical storms or other natural disasters (e.g., flooding) (51).

## Future Directions and Challenges

### Future Directions and Implications for the Risk of Introduction

#### Assessing the Risk of Introduction of JEV

Several epidemiological studies have been conducted to quantify vector and host parameters and to evaluate the risk of emergence of JEV in the US (5, 7–9, 52, 53). Risk assessment, as a decision tool, is a method to make decisions under uncertainty (54). This implies that approximations and assumptions often need to be made using data that are available, rather than ideal data (55).

The risk of introduction of JEV in the US, evaluated using a risk assessment framework, through infected adult mosquito vectors was predicted to be very high: there is a 0.95 median probability (95% CI: 0.80–0.99) of at least one infected mosquito, and a median of three infected mosquitoes (95% CI: 1–7), being introduced during March to October via aircraft, the most likely pathway of entry, to the US from JEV-affected countries (52). Mediterranean California and Eastern Temperate Forests ecoregions (covering all US states on the East Coast, except Southern Florida, the Midwest, and the Southeast), which are similar to the ecosystems found among the regions at risk, were the areas in the US with the highest risk of JEV introduction via infected mosquitoes transported in aircraft (52).

When considering other pathways of entry (e.g., birds, hosts, vaccines, other biologicals), the risk of JEV introduction was considered negligible. The risk of transmission was considered variable and the risk of establishment negligible given current conditions (53). Changing aspects and preconditions related to the introduction and transmission of JEV will also imply a change in probability estimation. Thus, revisiting the pathways of introduction and considering paths that were previously deemed as non-important (e.g., domestic and wild pigs) can lead to different assumptions and therefore, different probability estimates.

As discussed elsewhere (53), bird migration (e.g., flyways coming from Asia into the US through Alaska) was considered a negligible pathway for JEV introduction into the US. Short viremia in avian hosts [2-4 days (56)] and their long migration flights, life-long immunity after infection, the low probability of co-occurrence of an infectious migrant bird with competent vectors and susceptible birds, low number of competent vectors (e.g., *Aedes vexans*) in Alaska, where flyways coming from Asia and heading south to the US overlap, and Alaska's short mosquito season, are factors contributing to the dismissal of this pathway for JEV to enter and establish in the US (17, 53). When disregarding the entry of viremic migratory birds as a potential pathway of introduction for JEV, we may have not considered the role of climate change and land perturbation, which could push birds toward new habitats, with new mosquito vectors, and modulate pathogen dynamics.

Legal and illegal importation of potentially infected birds was deemed not important. Legal import of birds is regulated through the U.S. Department of Agriculture, Animal and Plant Health Inspection Service and the U.S. Fish and Wildlife Service. Although quarantine procedures are unlikely to support virus transmission to mosquitoes and to other birds, illegally imported birds, if infected, not subjected to quarantine or examination would be more likely to transmit the virus to mosquitoes and other birds (57).

It is important to note that all pathways of JEV introduction assessed in both the qualitative and quantitative risk assessment models (52, 53) pertained to inadvertent and intentional sources. Despite being considered of low, negligible or unknown risk, most intentional (e.g., illegal importation of animals) causes should not be disregarded. However, the scarcity and uncertainty of empirical data on movement of increasing populations of potentially infected competent vertebrate host animals (e.g., feral swine or ardeid birds) or illegal importation of animals, make these routes extremely challenging to be examined (51, 58).

#### Parallels With Other Arboviruses

Bluetongue virus, and Venezuelan equine encephalitis virus are examples of arboviruses whose emergence has been associated with the dispersal of vector species, introduction of animal hosts, climate effects, urbanization and globalization, among other factors (49). Similarly, WNV, a closely related flavivirus, was introduced to and became endemic in North America over a period of a few years (59). During the summer of 1999, several *Culex pipiens* complex mosquitoes were identified as the principal vector and house sparrows as important maintenance hosts (60–64). American crows (and some other corvids) suffered fulminating systemic disease and were deemed critical amplifying hosts (65–69). Previous experience in temperate regions of Europe suggested that introduced strains of WNV from Africa or the Mediterranean did not persist, and re-introduction was necessary for repeated outbreaks of disease (70); however, WNV is now endemic in Europe (as well as in the Middle East, Africa, Asia and Australia) (71). In North America, where WNV is also endemic, virus persistence was achieved, and sustained by long-term infections of both mosquitoes and birds (72, 73). Factors such as normal migration and legal or illegal importation of zoo, pet, domestic, or wild birds have been hypothesized to have played a role in the introduction of the WNV to the western hemisphere, whereas complex ecological factors determined its geographic spread (57). It is important to note that the North American introduction and establishment of the WNV overcame similar unfavorable circumstances to the ones faced with JEV, given sufficient time and introduction opportunities.

### Challenges

The recent decline in overall arboviral surveillance capacity (and lack of JEV surveillance in particular) in the US can compromise our ability to rapidly detect and respond to existing and emerging threats (74). There have been 14 travel-associated JE human cases reported among US citizens from 1973 to 2008, with cases most likely being acquired in Thailand, the Philippines, Vietnam, Singapore, Japan and China (75). Cases occurred among military personnel, tourists visiting friends and relatives, and expatriates. Since then, two additional cases were recorded, one fatal case in a US child that visited the Philippines, and a refugee traveling from Thailand to the US (76). All cases, thus far, have been imported.

Despite an estimated high risk of entry into the US via infected adult mosquitoes by aircraft (52, 53), no evidence of JEV emergence, transmission, or establishment has been reported up until now in the US *under current conditions* related to virus, vector, host and environment. Potential hypotheses for explaining the non-emergence of JEV in the US include: (1) the fragility of JEV in the environment, which is easily destroyed by heat, UV light and common detergents (52, 77); (2) potentially low mosquito distribution and host density in airport and seaport areas (which are considered the most likely pathways of US introduction) (52, 53, 78); (3) short apparent periods of viremia in pigs and ardeid birds, ranging from 3–4 days (56, 79); (4) insufficient contact rates between hosts and vectors; (5) cross-protection of JEV with other endemic flaviviruses, such as WNV and St. Louis encephalitis virus; and (6) a potentially limited infection capacity of mosquitoes during establishment.

Co-circulation and strain displacement are not new to flaviviruses as they have occurred in multiple areas for dengue virus and in the US for WNV (80–83). Gould et al. (50) speculated that given the vectors' widespread geographic range and high adaptability toward changing environmental conditions, another genotype could emerge in new regions (50). Similarly, the possible movement of vectors and hosts associated with urbanization, carried by tropical storms, or other natural disasters, could increase rates of contact and hence, transmission potential.

Future genotype displacement or genetic modifications can compromise current cross-protection, and in turn threaten vaccine effectiveness, current immunization and other public health programs (12). Other challenges associated with emergence or reemergence of JEV genotypes could include changes in transmission paths, disease burden, or host demographics (11, 84).

Although viremia in the amplifying host is short, recent studies pointing at transmission via oronasal secretions between pigs without the involvement of vectors (39), suggest a previously unrecognized mechanism of transmission may exist. Incomplete knowledge regarding JEV transmission in wild and domestic pigs may cause the role of these species in the epidemiology of JEV to be underestimated.

Japanese encephalitis is a vaccine-preventable disease, but recent research suggests that currently available vaccines (both inactivated and attenuated) may not provide complete protection against GV infection (85). Additionally, and because humans are dead-end hosts, JEV vaccination does not provide herd immunity (3). Whether or not new vaccines are needed to deal with this challenge is still under debate. Moreover, the introduction of JEV could have devastating public health consequences, especially in locations with naïve and aging populations such as in the US, usually affected by chronic diseases (immunocompromised population), where there is potentially no herd immunity against JEV. In addition to vaccines, reducing contact between mosquito vectors with humans and animal reservoirs would limit the duration and extent of viral outbreaks in the environment (5). In JE endemic countries, larval habitat treatment of rice fields by chemical or mechanical manipulation (86, 87) and adult aerial spraying (88) are the main methods used for management of mosquito vectors; these methods are also used by mosquito and vector control districts throughout the US. The public perception of the health and environmental effects associated with the use of pesticides, however, has greatly impacted the area coverage and the type of products used for mosquito mitigation. Larval habitat treatments with *Bacillus thuringiensis israelensis*, spinosad, and other dipteran-specific larvicides are largely unimpacted, but adulticidal treatments are heavily regulated by US state and federal agencies. States like California limit the application of some adulticide active ingredients in riparian zones (e.g., coastal marshes) where endangered species are found. Mosquito vector control districts in this state must consult the Pesticide Regulation's Endangered Species Custom Realtime Internet Bulletin Engine or PRESCRIBE dataset, prior to pesticide application in public areas, however, pesticide application in residential areas does not have such restrictions (89). These limitations could make proper and timely mitigation of vectors very difficult.

Although JEV has not established in the US, the conditions are rapidly changing. Reduced mosquito control in areas at highest risk (i.e., west coast), no active surveillance for JEV in place, increasing populations of vector species and host reservoirs, and emerging viral genotypes that may change the probability of establishment, may dictate the future emergence, and subsequent spread of JEV in the US. Similarly, the increase in population density and in human and animal movement, coupled with climate effects, habitat modification and other anthropogenic factors, emphasize the need for early detection of arboviral diseases through surveillance in areas at higher risk. Hence, we propose monitoring changes in host or vector population composition or dynamics, and/or environmental configuration that can be beneficial for virus introduction, in US areas at higher risk. Similarly, ongoing identification of emerging disease risks through surveillance (e.g., detection of virus in vectors and hosts) efforts will increase the speed by which US officials can detect pathogen emergence. Rapid response to outbreaks can be achieved by increasing preparedness efforts including education of citizens (e.g., through citizen science campaigns), clinicians and laboratory diagnosticians on disease recognition and prevention, and improvement of laboratory detection capabilities. Lastly, conducting an economic assessment linking disease risk at the wildlife-livestock interface and comparing the benefits and costs of risk management (e.g., surveillance, biosecurity) in both livestock and wildlife, as well as determining where public health efforts are required, can reduce the vulnerability and potential consequences of a JEV incursion in the US. The potential impact of the emergence of arboviral diseases, in particular JEV, a disease with high morbidity and mortality rates in humans, in a susceptible region such as the Americas and the US specifically, which has an increasingly globalized commerce and tourism as well as concentrated and interconnected livestock production, is large and can lead to long lasting effects on public health, economies, and production systems.

## Author Contributions

All authors listed have made a substantial, direct and intellectual contribution to the work, and approved it for publication.

### Conflict of Interest

The authors declare that the research was conducted in the absence of any commercial or financial relationships that could be construed as a potential conflict of interest. The handling Editor declared a shared affiliation, though no other collaboration, with several of the authors LC, LN, DM, and DSM.

## Footnotes

^1^Infection rate being defined as the sum of individual mosquitoes that test positive for JEV (or pools of mosquitoes, if applicable) divided by the total number of mosquitoes (or pools) tested (9).

^2^Transmission rate is the proportion of mosquitoes that were orally exposed to JEV and transmitted the virus on refeeding or contained the virus in their saliva or salivary glands (9, 20).

^3^Global Invasive Species Database (GISD). Invasive Species Specialist Group. Available online at: http://www.iucngisd.org/gisd/species.php?sc=73 (accessed September 26, 2019).
